# Comparative study between a single unit system and a two-unit cold standby system with varying demand

**DOI:** 10.1186/s40064-015-1484-7

**Published:** 2015-11-19

**Authors:** Reetu Malhotra, Gulshan Taneja

**Affiliations:** Department of Applied Sciences, Chitkara University, Rajpura, 140401 Punjab India; Department of Mathematics, M.D.U, Rohtak, 124001 Haryana India

**Keywords:** Stochastic model, Cable manufacturing plant, Single unit system, Two unit cold standby, Varying demand, Semi-Markov process, Regenerative point technique, 97K60, 90B25, 60K10

## Abstract

The concerned paper illustrates the comparison of two stochastic models of a cable manufacturing plant with varying demand. Here, it shows the comparison between a single unit system (Model 1) and a two-unit cold standby system (Model 2). In Model 1, the system is either in working state on some demand or put to shut down mode on no demand. In Model 2, at initial stage, one of the units is operative while the other is kept as cold standby. At times when the operative unit stops working due to some breakdown/failure, the standby unit instantaneously becomes operative while the repairman repairs the failed unit. In this working model, only one unit remains operative at a time. However, there may be a state when both the units fail. The comparison of systems is done by means of MTSF (mean time to system failure), steady state availability and profit function using Laplace transforms and software package Code-Blocks 13.12. Different graphs have been plotted to discover which model is superior to the other model under the given conditions. The system is analysed by making use of semi-Markov processes and regenerative point technique.

## Background

The manufacturing of tools and special equipments is an inevitable part of our modern society. When equipment fails, production falls instantaneously. To maintain production, one has to keep tools/equipment available at all times, in order to run the systems. Since this can be expensive, availability and profit of an industrial system are becoming increasingly important. Indeed, profit will increase when the availability of a system increases.

System reliability has also been one of the major factors in most of the system performance-related studies. Though many researchers made meaningful contributions a lot in the field of system reliability modelling, fewer studies have reported the comparative analysis of different types of systems. Tuteja et al. (1991), Alidrisi (1992), Mokaddis et al. (1994), Pan (1997), Chandrasekhar et al. (2004) and Xu et al. (2005) analysed the reliability and availability of standby systems by studying various parameter viz. partial failures, perfect or imperfect switching, Erlangian repair time and three types of repair facilities. Taneja and Naveen (2003), Ke and Chu (2007), Wang and Chen (2009) and Yusuf (2014) compared two models considering different situations such as expert repairman, redundant repairable system and switching failures. Zhang et al. (2012) and Dessie (2014) studied the modeling of diesel system in locomotives and HIV/AIDS dynamic evolution.

The demand has been kept fixed in most of these studies systems. However, in some practical situations there may be fluctuation in demand, such as the General Cable Energy System, where demand of the product varies.

General cable energy system (Taneja and Malhotra 2013) is a cable manufacturing plant where different types of cables are produced. Two extruders of diameter 65 and 120 mm are available which are put to operation on the basis of demand. Hence, variation in demand plays an important role in the functioning of such systems. Malhotra and Taneja (2013a, b) studied the cost-benefit analysis of a single unit system (Model 1) while introducing variation in demand. In this model, initially demand is greater than or equal to the production. If the operative unit ceases working, a repairman repairs the failed unit. If there is fall off in demand, the system moves to a state in which demand is less than the production. Moreover, if demand declines further, the system will be in a shut down state. Malhotra and Taneja (2015) compared two single units with varying demand. Malhotra and Taneja (2014) developed a model for a two- unit cold standby system, without considering a shut down state where both the units may become operative simultaneously depending upon the demand.

In practical situations however, out of the two units being studied (in two-unit cold standby systems), only one unit remains operative at a time and the other unit is kept standby. Standby unit works only when the first unit fails. Information of such systems was collected on visiting a cable manufacturing plant in H.P., India and the authors developed a new model (Model 2) in which besides studying the above behavior, a concept of a new state generated was observed when there is no demand and system goes in shut down mode.

Depending on the situation, a model can be better or worse, therefore the comparative study becomes more important. Taking this into consideration, comparison is done graphically between the concerned models (Models 1 and 2) by computing various measures of system effectiveness using Laplace transforms and software package Code-Blocks 13.12.

## Methods

The probabilistic analysis of the two models is analyzed by making use of semi-Markov processes and regenerative point technique.

### Notations used for the description of models

Op: Unit is in operative state
d ≥ p, d < p: Demand is not less than production, demand is less than production
C_S_, D: Unit is in cold standby state, down unit
F_r_/F_w_: Failed unit is under repair/waiting for repair
F_R_: Repair of failed unit continuing from previous state
λ, α: Failure rate, repair rate of the operative unit
γ_1_: Rate of decrease of demand < production
γ_2_: Rate of increase of demand ≥ production
γ_3_: Rate of going from upstate to downstate
γ_4_, β_1_: Rate of reaching the state of no demand from some demand in Model 1, Model 2
p_1_: Probability that during the repair time demand ≥ production
p_2_: Probability that during the repair time demand < production
ϕ_i_(t): c.d.f. of first passage time from regenerative state i to a failed state
AD*i*, AP*i*: Availability that the system is in upstate when demand ≥ production and when demand < production for each Model *i* where *i* = 1, 2
B*i*: Busy period analysis of the repairman for each Model *i* where *i* = 1, 2
V*i*: Expected number of visits of repairman for each Model *i* where *i* = 1, 2
DT*i*: Expected down time for each Model *i* where *i* = 1,2
P1: Profit incurred to the system for Model 1
NP2: Net profit [total profit incurred in Model 2—installation cost for addition unit (ICA)]
μ_i_: Mean sojourn time in regenerative state i before transiting to any other state
*: Symbol for Laplace transforms
**: Symbol for Laplace Stieltjes transforms
q_ij_(t), Q_ij_(t), p.d.f and c.d.f of first passage time from a regenerative state i to a regenerative state j or to a failed state j without visiting any other regenerative state in (0, t]
g(t), G(t): p.d.f. and c.d.f. of repair time for the unit

#### Description of Model 2

**Fig. 1:**
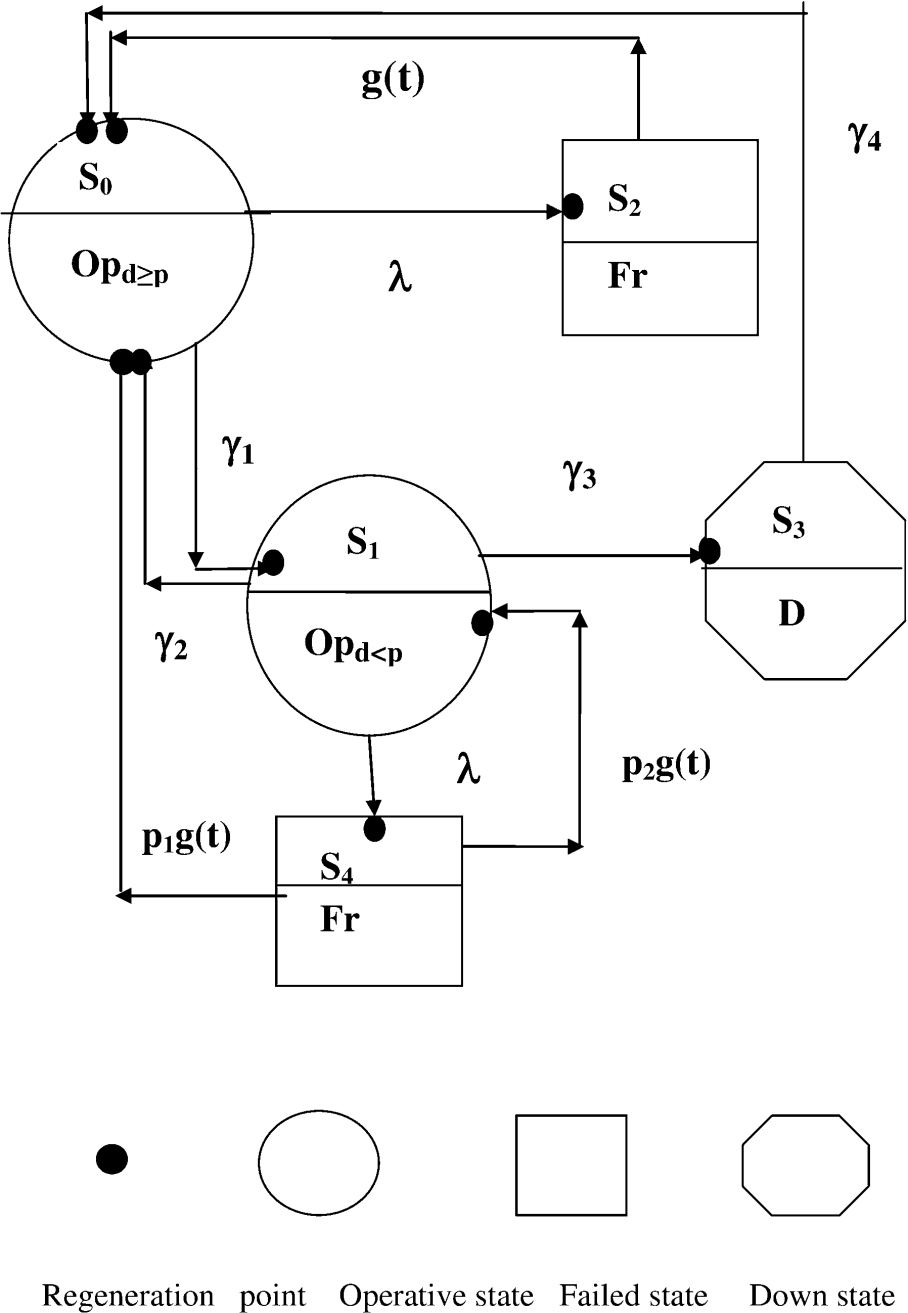
Transition state diagram of Model 1

The transition diagram shows the various states of the system in Fig. 1. In this diagram, S_0_, S_1_, S_2_, S_6_ and S_8_ are regenerative states. S_3_, S_7_ and S_9_ are non-regenerative states. States S_4_ and S_5_ are failed states.

**Fig. 2:**
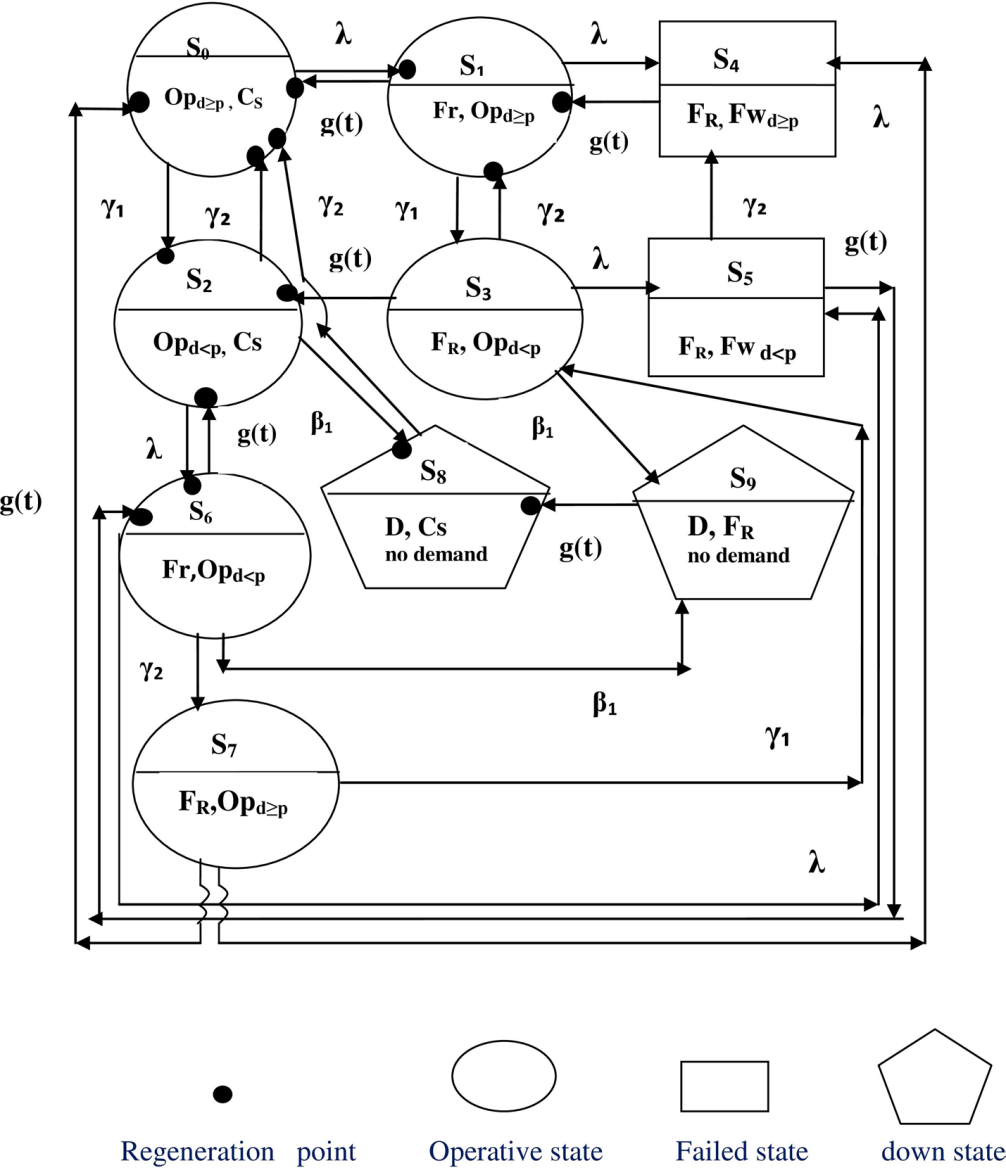
Transition state diagram of Model 2

In Model 2 (Fig. 2), initially (state S_0_) demand is not less than the production and one of the units is operative while the other is kept as cold standby. If the operative unit stops working, repairman repairs the failed unit and standby unit becomes operative instantaneously (state S_1_). When both the units are not working i.e. one is under repair and other is waiting to be repaired, the system will stay in the failed state S_4_. If demand gets decreased, system goes to state S_2_. After this state, three possibilities are there: (1) if demand gets increased, system goes back to state S_0_, (2) if demand further gets decreased, system goes to down state S_8_ and (3) if demand remains constant but operative unit fails, system goes to state S_6_. It moves to state S_7_ as the demand increased and failed unit is being repaired. From state S_3_ four possibilities are there (1) if failed unit gets repaired, then system moves to state S_2_, (2) if other unit also gets failed, system moves to state S_5_ (one unit is under repair and other is waiting to get repaired, (3) if demand gets increased, system goes back to state S_1_ and (4) if demand gets decreased further, system put to shut down state S_9_.

**Fig. 3:**
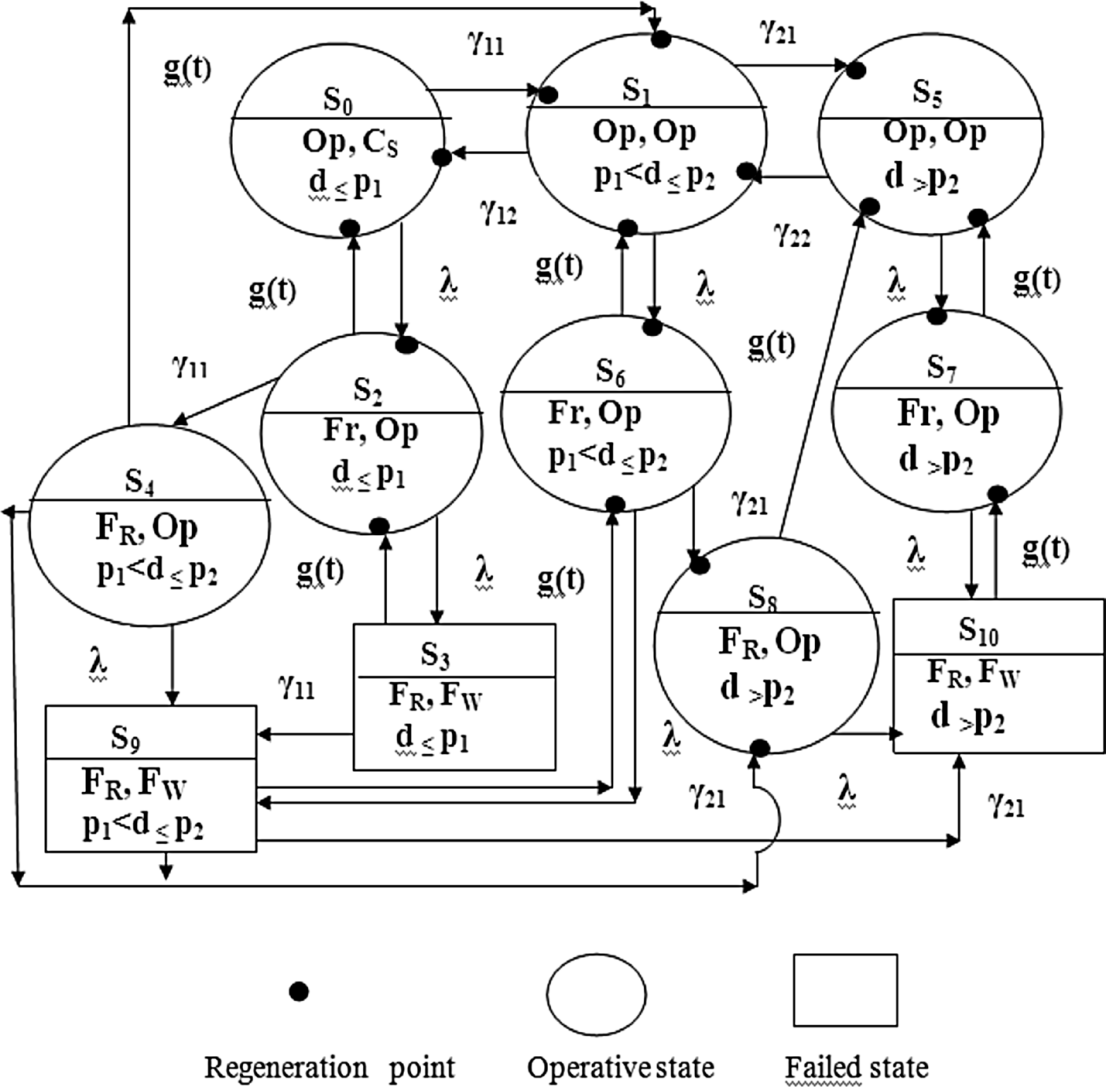
Transition state diagram of Model 3

Model 2 differs from the model presented in Fig. 3 (Malhotra and Taneja 2014) because the earlier paper did not include the case of shut down on very less or no demand. Also, in the earlier paper, the two units may become operative simultaneously when the demand is very high leading to increase in the failure rate of the system as compared to Model 2 but on the other hand high demand is met sooner in the earlier model. So, a comparative study between these two models is also reported here in the conclusion revealing which and when one model is better than the other.

Various assumptions for the Model 2 are as follows:

1. The units are similar and statistically independent.
2. Once the down state is reached, system will not be operative till all the units become operable, irrespective of the nature of demand.
3. A single repair facility is available. Each unit is new after repair.
4. All the random variables are independent. Switching is perfect and instantaneous.
5. Each unit is assumed to have an exponential distribution of the time to failure while the distribution of repair time is taken as arbitrary.

#### Measures of system effectiveness of Model 2

Using semi-Markov processes and regenerative point technique, various recursive relations are solved and measures of system effectiveness for Model 2 are evaluated. From transition probabilities q_ij_, the steady state probabilities pij of reaching different states and mean sojourn times have also been computed. The non-zero elements p_ij_ obtained as

1 $$p_{ij} = \mathop {\lim }\limits_{s \to 0} \,q_{{_{ij} }}^{*} (s)$$

To determine the mean time to system failure (MTSF) of the system, we regard the failed states as absorbing states. By probabilistic arguments, we obtain the following recursive relations for ϕ_i_ (t) where i = 0, 1, 2, 6, 8:

$$\phi_{0} (t) = Q_{01} (t)\;{\circledS }\;\phi_{0} (t) + Q_{02} (t)\;{\circledS }\;\phi_{0} (t)$$

$$\begin{aligned} \phi_{1} (t) &= Q_{10} (t)\;{\circledS }\;\phi_{0} (t) + Q_{11}^{3} (t)\;{\circledS }\;\phi_{1} (t) + Q_{{_{12} }}^{3} (t)\;{\circledS }\;\phi_{2} (t) \\ & \quad + Q_{18}^{(3,9)} (t)\;{\circledS }\;\phi_{8} (t) + Q_{15}^{3} (t) + Q_{14} (t) \\ \end{aligned}$$

$$\phi_{2} (t) = Q_{20} (t)\;{\circledS }\;\phi_{0} (t) + Q_{26} (t)\;{\circledS }\;\phi_{6} (t) + Q_{28} (t)\;{\circledS }\;\phi_{8} (t)$$

$$\begin{aligned} \phi_{6} (t) &= Q_{{_{60} }}^{7} (t)\;{\circledS }\;\phi_{0} (t) + Q_{61}^{(7,3)} (t)\;{\circledS }\;\phi_{1} (t) + Q_{62} (t)\;{\circledS }\;\phi_{2} (t) + Q_{62}^{(7,3)} (t)\;{\circledS }\;\phi_{2} (t) \\ & \quad+ Q_{68}^{(7,3,9)} (t)\;{\circledS }\;\phi_{8} (t) + Q_{{_{65} }}^{(7,3)} (t)\;{\circledS }\;Q_{64}^{7} (t) + Q_{62} (t) \\ \end{aligned}$$

$$\phi {}_{8}(t) = Q_{80} (t)\;{\circledS }\;\phi_{0} (t)$$

Taking Laplace–Steltjes Transform (L.S.T.) of these relations and solving them for $$\phi_{0}^{**} (s)$$, we obtain

2 $$\phi_{0}^{**} (s) = \frac{N(s)}{D(s)}$$

The reliability R(t) of the system at time t is given as

3 $$R\left( t \right) \, = Inverse \, Laplace \, transform \, of\,\frac{{1 - \phi_{0}^{**} (s)}}{s}$$

Now, the mean time to system failure (MTSF) when the system starts from the state ‘0’ is

4 $$MTSF = \int\limits_{0}^{\infty } {R\left( t \right)dt} = \mathop {\lim }\limits_{s \to 0} R^{*} \left( s \right)$$

Using L' Hospital rule and putting the value of $$\phi_{0}^{**} (s)$$ from Eq. (2), we have

5 $${\text{MTSF }}\left( {\text{M2}} \right) = N/D$$

where

$$\begin{aligned} N &= \mu_{0} \left( {\left(1 - p_{11}^{3} \right)\left( {1 - p_{26} \left(p_{62} + p_{62}^{(7,3)} \right)} \right) - p_{26} p_{12}^{3} p_{61}^{(7,3)} } \right) + \mu_{1} \left( {p_{01} + p_{61}^{(7,3)} p_{26} } \right. \\ & \quad - \left. {p_{01} p_{26} \left( {p_{62} + p_{61}^{(7,3)} + p_{62}^{(7,3)} } \right)} \right) + \left( {p_{01} p_{12}^{3} + p_{02} \left(1 - p_{11}^{3} \right)} \right)\left( {\mu_{2} + p_{26} \mu_{6} } \right. \\ & \quad + \left( {p_{26} \left( {p_{68}^{9} + p_{68}^{(7,3,9)} + p_{28} } \right)\mu_{8} } \right) + p_{18}^{(3,9)} \left( {p_{01} \left( {1 - p_{26} \left( {p_{62} + p_{62}^{(7,3)} } \right)} \right) - p_{26} p_{02} p_{61}^{(7,3)} } \right)\mu_{8} \\ D &= - \left( {p_{26} \left( {p_{68}^{9} + p_{68}^{(7,3,9)} + p_{28} } \right)\left( {p_{01} p_{12}^{3} + p_{02} \left( {1 - p_{11}^{3} } \right)} \right) - p_{18}^{(3,9)} \left( {p_{01} \left( {1 - p_{26} \left( {p_{62} + p_{62}^{(7,3)} } \right)} \right)} \right.} \right. \\ & \quad - \left. {p_{26} p_{02} p_{61}^{(7,3)} } \right) - \left( {p_{26} \left( {p_{62} + p_{62}^{(7,3)} } \right)\left( {1 - p_{11}^{3} } \right) + p_{01} p_{10} } \right) + p_{26} \left( {p_{12}^{3} \left( { - p_{61}^{(7,3)} - p_{01} p_{60}^{7} } \right)} \right. \\ & \quad - \left. {p_{02} p_{10} p_{61}^{(7,3)} } \right) + \left( {1 - p_{11}^{3} } \right)\left( { - p_{26} p_{02} p_{60}^{7} - p_{02} p_{20} + 1} \right) - \left. {p_{01} p_{10} - p_{01} p_{20} p_{12}^{3} } \right) \\ \end{aligned}$$

Letting AD2(t) as the probability that the system is in upstate when demand is not less than production at instant t given that it entered the state i at time t. AP2(t) as the probability that the system is in upstate when demand is less than production at time t. B2(t) as the probability that the repairman is busy to repair the failed unit at instant t given that it entered the regenerative state i at any time t. V2(t) as the expected number of visits by the server in (0, t] given that the system entered the regenerative state i. DT2(t) as the probability that the system is in down state at instant t given that the system entered regenerative state i at any time t.

These measures of system effectiveness (AD2, AP2, B2, V2, DT2) have been obtained, in steady state, by using the probabilistic arguments in the similar fashion as shown for MTSF except the fact that here the failed state is not considered as the absorbing state. For more elaboration of these measures, Malhotra and Taneja (2014) may be referred to.

6 $$\begin{aligned} {\text{Thus}},\,{\text{Expected total revenue}}\, & = \,\left( {{\text{Revenue}}/{\text{time when d }} \ge {\text{ p }}\left( {{\text{C}}_{0} } \right)} \right)*{\text{steady state availability }}\left( {\text{AD2}} \right) \\ & \quad + \, \left( {{\text{revenue}}/{\text{time when d }} < {\text{ p }}\left( {{\text{C}}_{ 1} } \right)} \right)*{\text{steady state availability }}\left( {\text{AP2}} \right) \\ & = {\text{C}}_{0} *{\text{ AD2}} + {\text{ C}}_{ 1} *{\text{AP2}} \\ \end{aligned}$$

7 $$\begin{aligned} {\text{Expected total cost}}\, & = \,({\text{Cost }}({\text{C}}_{ 2} )/{\text{time for engaging the repairman for repair}})*{\text{busy Period }}\left( {\text{B2}} \right) \\ & \quad + \,({\text{cost }}({\text{C}}_{ 3} ){\text{per visit of the repairman}})*{\text{expected number of visits }}\left( {\text{V2}} \right) \\ & \quad + \,({\text{loss }}({\text{C}}_{ 4} )/{\text{time during system remains down}})*{\text{expected down time }}\left( {\text{DT2}} \right) \\ & = \,{\text{C}}_{ 2} *{\text{ B2}} + {\text{C}}_{ 3} *{\text{ V2}} + {\text{ C}}_{ 4} *{\text{ DT2}} \\ \end{aligned}$$

Expected total profit = Expected total revenue − expected total cost is, therefore, given by

8 $$Profit \, \left( {\text{NP2}} \right) = \left( {{\text{C}}_{0} * {\text{AD2}} + {\text{C}}_{ 1} * {\text{AP2}}} \right) - \left( {{\text{C}}_{ 2} * {\text{B2}} + {\text{C}}_{ 3} * {\text{V2}} + {\text{C}}_{ 4} * {\text{DT2}}} \right)$$

where $${\text{AD2}} = \frac{{N_{1} }}{{D_{2} }},\;{\text{AP2 }} = \frac{{N_{2} }}{{D_{2} }},\;{\text{B2}} = \frac{{N_{3} }}{{D_{2} }},{\text{V2}} = \frac{{N_{4} }}{{D_{2} }},\;{\text{DT2}} = \frac{{N_{5} }}{{D_{2} }}$$.

9 $$\begin{aligned} N_{I} &= \mu_{0} \left( {\left( {1 - \left( {p_{66}^{5} + p_{66}^{(7,3,5)} } \right) - p_{26} \left( {p_{62} + p_{62}^{(7,3)} } \right)} \right)\left( {1 - \left( {p_{11}^{3} + p_{11}^{4} + p_{11}^{(3,5,4)} } \right)} \right) - \left( {p_{61}^{(7,3)} + p_{61}^{(7,4)} } \right.} \right. \\ & \quad +\left. {\left. {p_{61}^{(7,3,5,4)} + p_{61}^{(5,4)} } \right)\left( {p_{12}^{3} p_{26} + p_{16}^{(3,5)} } \right)} \right) + \left( {\frac{{\gamma_{1} }}{{(\beta_{1} + \gamma_{2} - \gamma_{1} )}}(\mu_{1} - \mu_{6} ) + \mu_{1} } \right) \\ & \quad\times \left( {p_{01} \left( {1 - \left( {p_{66}^{5} + p_{66}^{(7,3,5)} } \right) - p_{26} \left( {p_{62} + p_{62}^{(7,3)} } \right)} \right) + p_{26} p_{02} \left( {p_{61}^{(7,3)} + p_{61}^{(7,4)} + p_{61}^{(7,3,5,4)} + p_{61}^{(5,4)} } \right)} \right) \\ \end{aligned}$$

10 $$\begin{aligned} N_{2} & = \mu _{2} \left( {\left( {p_{{02}} \left( {1 - \left( {p_{{11}}^{3} + p_{{11}}^{4} + p_{{11}}^{{(3,5,4)}} } \right)} \right) + p_{{01}} p_{{12}}^{3} } \right)\left( {1 - \left( {p_{{66}}^{5} + p_{{66}}^{{(7,3,5)}} } \right)} \right)} \right.\;{\kern 1pt} \\ & \left. {\quad +\, p_{{01}} p_{{16}}^{{(3,5)}} \left( {p_{{62}} + p_{{62}}^{{(7,3)}} } \right) - p_{{02}} p_{{16}}^{{(3,5)}} \left( {p_{{61}}^{{(7,3)}} + p_{{61}}^{{(7,4)}} + p_{{61}}^{{(7,3,5,4)}} + p_{{61}}^{{(5,4)}} } \right)} \right) \\ & \quad + \left( {\mu _{6} + \frac{{(\mu _{1} - \mu _{6} )}}{{(\beta _{1} + \gamma _{2} - \gamma _{1} )}}\left( {\gamma _{2} + \frac{1}{{(\beta _{1} + \gamma _{2} - \gamma _{1} )}}} \right)} \right)\; \\ {\kern 1pt} & \quad - \frac{{\gamma _{1} \gamma _{2} }}{{(\lambda + \beta _{1} + \gamma _{2} )}}(\beta _{1} + \gamma _{2} - \gamma _{1} )(\mu _{6} - k_{2} )\mu _{2} \\ & \quad \times \left( {p_{{01}} \left( {p_{{16}}^{{(3,5)}} + p_{{26}} p_{{12}}^{3} } \right) + p_{{26}} p_{{02}} \left( {1 - \left( {p_{{11}}^{3} + p_{{11}}^{4} + p_{{11}}^{{(3,5,4)}} } \right)} \right)} \right) \\ \end{aligned}$$

11 $$\begin{aligned} D_{2} & = \mu _{0} \left( {\left( {1 - \left( {p_{{66}}^{5} + p_{{66}}^{{(7,3,5)}} } \right) - p_{{26}} \left( {p_{{62}} + p_{{62}}^{{(7,3)}} } \right)} \right)\left( {1 - \left( {p_{{11}}^{3} + p_{{11}}^{4} + p_{{11}}^{{(3,5,4)}} } \right)} \right)} \right. \\ & \quad - \left( {p_{{61}}^{{(7,3)}} + p_{{61}}^{{(7,4)}} } \right.\;{\kern 1pt} + \left. {\left. {p_{{61}}^{{(7,3,5,4)}} + p_{{61}}^{{(5,4)}} } \right)\left( {p_{{12}}^{3} p_{{26}} + p_{{16}}^{{(3,5)}} } \right)} \right) \\ & \quad + \varepsilon _{1} \left( {p_{{01}} \left( {1 - \left( {p_{{66}}^{5} + p_{{66}}^{{(7,3,5)}} } \right) - p_{{26}} \left( {p_{{62}} + p_{{62}}^{{(7,3)}} } \right)} \right)} \right. \\ & \quad + \left. {p_{{26}} p_{{02}} \left( {p_{{61}}^{{(7,3)}} + p_{{61}}^{{(7,4)}} + p_{{61}}^{{(7,3,5,4)}} + p_{{61}}^{{(5,4)}} } \right)} \right) \\ & \quad + \mu _{2} \left( {\left( {p_{{02}} \left( {1 - \left( {p_{{11}}^{3} + p_{{11}}^{4} + p_{{11}}^{{(3,5,4)}} } \right)} \right) + p_{{01}} p_{{12}}^{3} } \right)\left( {1 - \left( {p_{{66}}^{5} + p_{{66}}^{{(7,3,5)}} } \right)} \right)} \right. \\ & \quad \times p_{{01}} p_{{16}}^{{(3,5)}} \left( {p_{{62}} + p_{{62}}^{{(7,3)}} } \right) - p_{{02}} p_{{16}}^{{(3,5)}} \left( {p_{{61}}^{{(7,3)}} + p_{{61}}^{{(7,4)}} + p_{{61}}^{{(7,3,5,4)}} + p_{{61}}^{{(5,4)}} } \right) \\ & \quad + \varepsilon _{4} \;\;{\kern 1pt} \left( {p_{{01}} \left( {1 - \left( {p_{{66}}^{5} + p_{{66}}^{{(7,3,5)}} } \right) - p_{{26}} \left( {p_{{62}} + p_{{62}}^{{(7,3)}} } \right)} \right)} \right) \\ & \quad + \left( {p_{{01}} \left( {p_{{16}}^{{(3,5)}} + p_{{26}} p_{{12}}^{3} } \right) + p_{{26}} p_{{02}} \left( {1 - \left( {p_{{11}}^{3} + p_{{11}}^{4} + p_{{11}}^{{(3,5,4)}} } \right)} \right)} \right) \\ & \quad + \left( {p_{{01}} \left( {p_{{12}}^{3} p_{{28}} - p_{{18}}^{{(3,9)}} } \right)} \right.\;{\kern 1pt} + \left. {p_{{28}} p_{{02}} \left( {1 - \left( {p_{{11}}^{3} + p_{{11}}^{4} + p_{{11}}^{{(3,5,4)}} } \right)} \right)} \right) \\ & \quad + \mu _{8} \left( {p_{{26}} \left( {p_{{01}} p_{{12}}^{3} + p_{{02}} \left( {1 - \left( {p_{{11}}^{3} + p_{{11}}^{4} + p_{{11}}^{{(3,5,4)}} } \right)} \right)} \right) + p_{{01}} p_{{16}}^{{(3,5)}} } \right)\left( {p_{{68}}^{9} + p_{{68}}^{{(7,3,9)}} } \right) \\ & \quad \times \left( {1 - \left( {p_{{66}}^{5} + p_{{66}}^{{(7,3,5)}} } \right)} \right) + \left( {p_{{16}}^{{(3,5)}} p_{{28}} - p_{{18}}^{{(3,9)}} p_{{26}} } \right)\left( {p_{{01}} \left( {p_{{62}} + p_{{62}}^{{(7,3)}} } \right)} \right.\;\; - \left. {p_{{02}} \left( {p_{{68}}^{9} + p_{{68}}^{{(7,3,9)}} } \right)} \right) \\ \end{aligned}$$

12 $$\begin{aligned} N_{3} &= \frac{{\mu_{1} }}{{(\lambda + \gamma_{1} - \gamma_{2} )(\beta_{1} + \gamma_{2} - \gamma_{1} )(\lambda + \gamma_{1} )}}\left( {(\lambda + \gamma_{1} - \gamma_{2} )(\lambda \beta_{1} + \lambda \gamma_{2} + \gamma_{1} \gamma_{2} )} \right. \\ & \quad- \left. {\gamma_{1} (\lambda \gamma_{2} - \lambda^{2} - \lambda \gamma_{1} )} \right) + \frac{{\mu_{6} }}{{(\lambda + \beta_{1} + \gamma_{2} )(\beta_{1} + \gamma_{2} - \gamma_{1} )(\lambda + \beta_{1} )}}( - \gamma_{1} \gamma_{2} (\lambda + \beta_{1} )(\lambda + \gamma_{2} ) \\ &\quad + \lambda (\lambda + \beta_{1} + \gamma_{2} - 1)) + \frac{{k_{1} \gamma_{1} (\lambda + \beta_{1} )}}{{(\lambda + \beta_{1} + \gamma_{2} )(\lambda + \gamma_{1} )}}\left( {p_{01} \left( {1 - \left( {p_{66}^{5} + p_{66}^{(7,3,5)} } \right) - p_{26} \left( {p_{62} + p_{62}^{(7,3)} } \right)} \right)} \right. \\ & \quad+ \left. {p_{26} p_{02} \left( {p_{61}^{(7,3)} + p_{61}^{(7,4)} + p_{61}^{(7,3,5,4)} + p_{61}^{(5,4)} } \right)} \right) \\ & \quad + \frac{{\mu_{1} }}{{(\lambda + \gamma_{1} )(\beta_{1} + \gamma_{2} - \gamma_{1} )^{2} }}((\beta_{1} + \gamma_{2} - \gamma_{1} )((\lambda + \gamma_{1} )\gamma_{2} - \lambda \gamma_{1} ) + \gamma_{1} \gamma_{2} (\lambda - \beta_{1} )) \\ & \quad+ \frac{{\mu_{6} }}{{(\lambda + \gamma_{1} )(\beta_{1} + \gamma_{2} - \gamma_{1} )^{2} }}(((\beta_{1} - \gamma_{2} )\gamma_{1} \gamma_{2} + (\lambda + \gamma_{1} )(\beta_{1} + \gamma_{2} - \gamma_{1} )) - \frac{{\lambda \gamma_{1} \gamma_{2} }}{{(\lambda + \beta_{1} + \gamma_{2} )^{2} (\lambda + \gamma_{1} )}} \\ & \quad - \frac{1}{{(\lambda + \beta_{1} + \gamma_{2} )(\beta_{1} + \gamma_{2} - \gamma_{1} )(\lambda + \beta_{1} )}}((\beta_{1} (\beta_{1} + \gamma_{2} - \gamma_{1} ) + \lambda \gamma_{2} )(\lambda + \beta_{1} ) + \lambda \gamma_{2} (\beta_{1} + \gamma_{2} - \gamma_{1} ))) \\ & \quad + \frac{{(\mu_{6} - k_{2} )\gamma_{2} }}{{(\beta_{1} + \gamma_{2} - \gamma_{1} )(\lambda + \beta_{1} + \gamma_{2} )}} + \frac{\lambda }{{(\lambda + \beta_{1} )\gamma_{2} }}(1 - k_{3} )\\ & \quad \times \left( {p_{01} \left( {p_{12}^{3} p_{26} + p_{16}^{(3,5)} } \right) + p_{02} p_{26} \left( {1 - \left( {p_{11}^{3} + p_{11}^{4} + p_{11}^{(3,5,4)} } \right)} \right)} \right) \\ \end{aligned}$$

13 $$\begin{gathered} N_{4} = p_{01} \left( {1 - \left( {p_{11}^{3} + p_{11}^{4} + p_{11}^{(3,5,4)} } \right)\left( {1 - \left( {p_{66}^{5} + p_{66}^{(7,3,5)} } \right)} \right) - \left( {p_{66}^{5} + p_{66}^{(7,3,5)} } \right)\left( {p_{61}^{(7,3)} + p_{61}^{(7,4)} + p_{61}^{(7,3,5,4)} + p_{61}^{(5,4)} } \right)} \right. - p_{26} \left( {\left( {1 - \left( {p_{11}^{3} + p_{11}^{4} + p_{11}^{(3,5,4)} } \right)} \right)\left( {p_{62} + p_{62}^{(7,3)} } \right) - p_{16}^{(3,5)} \left( {p_{62} + p_{62}^{(7,3)} } \right) - p_{12}^{3} \left( {\left( {1 - \left( {p_{61}^{(7,3)} + p_{61}^{(7,4)} + p_{61}^{(7,3,5,4)} + p_{61}^{(5,4)} } \right)} \right)} \right.} \right. - \left. {\left. {\left. {\left( {p_{66}^{5} + p_{66}^{(7,3,5)} } \right)} \right)} \right)} \right) + p_{26} p_{02} \left( {1 - \left( {p_{11}^{3} + p_{11}^{4} + p_{11}^{(3,5,4)} } \right)\left( {1 - \left( {p_{66}^{5} + p_{66}^{(7,3,5)} } \right)} \right) - \left( {p_{66}^{5} + p_{66}^{(7,3,5)} } \right)} \right. - \left. {p_{16}^{(3,5)} \left( {p_{61}^{(7,3)} + p_{61}^{(7,4)} + p_{61}^{(7,3,5,4)} + p_{61}^{(5,4)} } \right)} \right) \end{gathered}$$

14 $$\begin{gathered} N_{5} = \mu_{8} \left( {\left( {p_{26} \left( {p_{01} p_{12}^{3} + p_{02} \left( {1 - \left( {p_{11}^{3} + p_{11}^{4} + p_{11}^{(3,5,4)} } \right)} \right)} \right) + p_{01} p_{16}^{(3,5)} } \right)\left( {p_{68}^{9} + p_{68}^{(7,3,9)} } \right) + \left( {p_{01} \left( {p_{12}^{3} p_{28} - p_{18}^{(3,9)} } \right)} \right.} \right. + \left. {p_{28} p_{02} \left( {1 - \left( {p_{11}^{3} + p_{11}^{4} + p_{11}^{(3,5,4)} } \right)} \right)} \right)\left( {1 - \left( {p_{66}^{5} + p_{66}^{(7,3,5)} } \right)} \right) + \left( {p_{16}^{(3,5)} p_{28} - p_{18}^{(3,9)} p_{26} } \right)\left( {p_{01} \left( {p_{62} + p_{62}^{(7,3)} } \right)} \right. - \left. {p_{02} \left( {p_{68}^{9} + p_{68}^{(7,3,9)} } \right)} \right) \\ \end{gathered}$$

where

15 $$p_{01} = \frac{\lambda }{{\left( {\lambda + \gamma_{1} } \right)}}$$

16 $$p_{02} = \frac{{\gamma_{1} }}{{\left( {\lambda + \gamma_{1} } \right)}}$$

17 $$p_{10} = g*(\lambda + \gamma_{1} )$$

18 $$p_{11}^{3} = \frac{{\gamma_{1} \gamma_{2} }}{{(\beta_{1} + \gamma_{2} - \gamma_{1} )}}\left[ {\left( {1 - \frac{{g*(\lambda + \gamma_{1} )}}{{(\lambda + \gamma_{1} )}}} \right) - \frac{{1 - g*(\lambda + \beta_{1} + \gamma_{2} ) \, }}{{(\lambda + \beta_{1} + \gamma_{2} )}}} \right]$$

19 $$p_{12}^{3} = \frac{{\gamma_{1} }}{{(\beta_{1} + \gamma_{2} - \gamma_{1} )}}\left( {g*(\lambda + \gamma_{1} ) - g*(\lambda + \beta_{1} + \gamma_{2} )} \right)$$

20 $$p_{11}^{4} = \lambda \left( {\frac{{1 - g*(\lambda + \gamma_{1} )}}{{(\lambda + \gamma_{1} )}}} \right)$$

21 $$p_{1,1}^{(3,5,4)} = \lambda \gamma_{1} \gamma_{2} \left( \begin{aligned} \frac{1}{{\gamma_{2} (\lambda + \gamma_{1} )\left( {\lambda + \beta_{1} + \gamma_{2} } \right)}} + \frac{{g*(\lambda + \gamma_{1} )}}{{(\beta_{1} + \gamma_{2} - \gamma_{1} )(\lambda + \gamma_{1} )\left( {\lambda + \gamma_{1} \gamma_{2} } \right)}} + \hfill \\ \frac{{g*\left( {\lambda + \beta_{1} + \gamma_{2} } \right)}}{{\left( {\lambda + \beta_{1} + \gamma_{2} } \right)(\beta_{1} + \gamma_{2} - \gamma_{1} )(\lambda + \beta_{1} )}} - \frac{{g*(\gamma_{2} )}}{{\gamma_{2} (\lambda + \beta_{1} )(\lambda + \gamma_{1} - \gamma_{2} )}} \hfill \\ \end{aligned} \right)$$

22 $$p_{13}^{5} = \frac{{\lambda \gamma_{1} }}{{\left( {\beta_{1} + \gamma_{2} - \gamma_{1} } \right)}}\left( { - \frac{{\left( {1 - g*(\lambda + \beta_{1} + \gamma_{2} )} \right)}}{{(\lambda + \beta_{1} + \gamma_{2} )}} + \frac{{\left( {1 - g*(\lambda + \gamma_{1} )} \right)}}{{(\lambda + \gamma_{1} )}}} \right)$$

23 $$p_{16}^{(3,5)} = \lambda \gamma_{1} \left( {\frac{{g*(\lambda + \beta_{1} + \gamma_{2} )}}{{\left( {\beta_{1} + \gamma_{2} - \gamma_{1} } \right)(\lambda + \beta_{1} )}} + \frac{{g*(\gamma_{2} )}}{{\left( {\lambda + \gamma_{1} - \gamma_{2} } \right)(\lambda + \beta_{1} )}} - \frac{{g*(\lambda + \gamma_{1} )}}{{\left( {\beta_{1} + \gamma_{2} - \gamma_{1} } \right)(\lambda + \gamma_{1} - \gamma_{2} )}}} \right)$$

24 $$p_{18}^{(3,9)} = \beta_{1} \gamma_{1} \left( {\frac{1}{{\left( {\lambda + \beta_{1} + \gamma_{2} } \right)(\lambda + \gamma_{1} )}} + \frac{{g*\left( {\lambda + \beta_{1} + \gamma_{2} } \right)}}{{\left( {\beta_{1} + \gamma_{2} - \gamma_{1} } \right)\left( {\lambda + \beta_{1} + \gamma_{2} } \right)}} - \frac{{g*(\lambda + \gamma_{1} )}}{{\left( {\beta_{1} + \gamma_{2} - \gamma_{1} } \right)(\lambda + \gamma_{1} )}}} \right)$$

25 $$p_{14} = \lambda \left( {\frac{{1 - g*(\lambda + \gamma_{1} )}}{{(\lambda + \gamma_{1} )}}} \right)$$

26 $$p_{20} = \frac{{\gamma_{2} }}{{(\lambda + \beta_{1} + \gamma_{2} )}}$$

27 $$p_{26} = \frac{\lambda }{{(\lambda + \beta_{1} + \gamma_{2} )}}$$

28 $$p_{28} = \frac{{\beta_{1} }}{{(\lambda + \beta_{1} + \gamma_{2} )}}$$

29 $$p_{60}^{7} = \frac{{\gamma_{2} }}{{\left( {\beta_{1} + \gamma_{2} - \gamma_{1} } \right)}}(g*(\lambda + \gamma_{1} ) - g*(\lambda + \beta_{1} + \gamma_{2} ))$$

30 $$p_{61}^{(5,4)} = \lambda \gamma_{2} \left( {\frac{1}{{\left( {\lambda + \beta_{1} + \gamma_{2} } \right)\gamma_{2} }} - \frac{{g*\left( {\gamma_{2} } \right)}}{{\gamma_{2} \left( {\lambda + \beta_{1} } \right)}} + \frac{{g*\left( {\lambda + \beta_{1} + \gamma_{2} } \right)}}{{\left( {\lambda + \beta_{1} + \gamma_{2} } \right)(\lambda + \beta_{1} )}}} \right)$$

31 $$p_{61}^{(7,3)} = \gamma_{2}^{2} \gamma_{1} \left( \begin{aligned} &\frac{1}{{(\lambda + \beta_{1} + \gamma_{2} )^{2} \left( {\lambda + \gamma_{1} } \right)}} + \frac{{g*(\lambda + \beta_{1} + \gamma_{2} )\left( {2\beta_{1} + 2\gamma_{2} + \lambda - \gamma_{1} } \right)}}{{\left( {\beta_{1} + \gamma_{2} - \gamma_{1} } \right)^{2} (\lambda + \beta_{1} + \gamma_{2} )^{2} }} \hfill \\ &- \frac{{g*^{\prime } (\lambda + \beta_{1} + \gamma_{2} )}}{{\left( {\beta_{1} + \gamma_{2} - \gamma_{1} } \right)(\lambda + \beta_{1} + \gamma_{2} )}} - \frac{{g*(\lambda + \gamma_{1} )}}{{\left( {\beta_{1} + \gamma_{2} - \gamma_{1} } \right)^{2} (\lambda + \gamma_{1} )}} \hfill \\ \end{aligned} \right)$$

32 $$p_{{61}}^{{(7,4)}} = \lambda \gamma _{2} \left( \begin{aligned} &\frac{1}{{\left( {\lambda + \beta _{1} + \gamma _{2} } \right)(\lambda + \gamma _{1} )}} + \frac{{g*\left( {\lambda + \beta _{1} + \gamma _{2} } \right)}}{{\left( {\beta _{1} + \gamma _{2} - \gamma _{1} } \right)\left( {\lambda + \beta _{1} + \gamma _{2} } \right)}} \hfill \\ &- \frac{{g*(\lambda + \gamma _{1} )}}{{\left( {\beta _{1} + \gamma _{2} - \gamma _{1} } \right)(\lambda + \gamma _{1} )}} \hfill \\ \end{aligned} \right)$$

33 $$\begin{aligned} p_{61}^{(7,3,5,4)} &= \lambda \gamma_{2}^{2} \gamma_{1} \left( {\frac{1}{{\gamma_{2} (\lambda + \beta_{1} + \gamma_{2} )^{2} \left( {\lambda + \gamma_{1} } \right)}} - \frac{{g*(\gamma_{2} )}}{{\gamma_{2} (\lambda + \gamma_{1} - \gamma_{2} )(\lambda + \beta_{1} )^{2} }}} \right. \\ &\quad + \frac{{g*(\lambda + \gamma_{1} )}}{{\left( {\beta_{1} + \gamma_{2} - \gamma_{1} } \right)^{2} (\lambda + \gamma_{1} )(\lambda + \gamma_{1} - \gamma_{2} )}} + g*(\lambda + \beta_{1} + \gamma_{2} ) \\ & \quad\times \left( {\frac{ - 1}{{\gamma_{2} (\lambda + \beta_{1} + \gamma_{2} )^{2} \left( {\lambda + \gamma_{1} } \right)}} + \frac{1}{{\gamma_{2} (\lambda + \beta_{1} )^{2} \left( {\lambda + \gamma_{1} - \gamma_{2} } \right)}}} \right. \\ & \quad- \left. {\frac{1}{{\left( {\beta_{1} + \gamma_{2} - \gamma_{1} } \right)^{2} (\lambda + \gamma_{1} )(\lambda + \gamma_{1} - \gamma_{2} )}}} \right) \\ &\quad - \left. {\frac{{g*^{\prime } (\lambda + \beta_{1} + \gamma_{2} )}}{{\left( {\beta_{1} + \gamma_{2} - \gamma_{1} } \right)(\lambda + \beta_{1} + \gamma_{2} )(\lambda + \beta_{1} )}}} \right) \\ \end{aligned}$$

34 $$p_{62} = g*(\lambda + \beta_{1} + \gamma_{2} )$$

35 $$p_{62}^{(7,3)} = \gamma_{2} \gamma_{1} \left( { - \frac{{g*(\lambda + \beta_{1} + \gamma_{2} )}}{{\left( {\beta_{1} + \gamma_{2} - \gamma_{1} } \right)^{2} }} + \frac{{g*^{\prime } (\lambda + \beta_{1} + \gamma_{2} )}}{{\left( {\beta_{1} + \gamma_{2} - \gamma_{1} } \right)}} + \frac{{g*(\lambda + \gamma_{1} )}}{{\left( {\beta_{1} + \gamma_{2} - \gamma_{1} } \right)^{2} }}} \right)$$

36 $$p_{64}^{7} = \frac{{\lambda \gamma_{2} }}{{\left( {\beta_{1} + \gamma_{2} - \gamma_{1} } \right)}}\left( { - \frac{{1 - g*\left( {\lambda + \beta_{1} + \gamma_{2} } \right)}}{{\left( {\lambda + \beta_{1} + \gamma_{2} } \right)}} + \frac{{1 - g*(\lambda + \gamma_{1} )}}{{(\lambda + \gamma_{1} )}}} \right)$$

37 $$p_{65} = \lambda \left( {\frac{{1 - g*\left( {\lambda + \beta_{1} + \gamma_{2} } \right)}}{{\left( {\lambda + \beta_{1} + \gamma_{2} } \right)}}} \right)$$

38 $$p_{65}^{(7,3)} = \lambda \gamma_{2} \gamma_{1} \left( \begin{aligned} &\frac{1}{{(\lambda + \beta_{1} + \gamma_{2} )^{2} \left( {\lambda + \gamma_{1} } \right)}} + \frac{{g*(\lambda + \beta_{1} + \gamma_{2} )\left( {2\beta_{1} + 2\gamma_{2} + \lambda - \gamma_{1} } \right)}}{{\left( {\beta_{1} + \gamma_{2} - \gamma_{1} } \right)^{2} (\lambda + \beta_{1} + \gamma_{2} )^{2} }} \hfill \\ &- \frac{{g*^{\prime } (\lambda + \beta_{1} + \gamma_{2} )}}{{\left( {\beta_{1} + \gamma_{2} - \gamma_{1} } \right)(\lambda + \beta_{1} + \gamma_{2} )}} - \frac{{g*(\lambda + \gamma_{1} )}}{{\left( {\beta_{1} + \gamma_{2} - \gamma_{1} } \right)^{2} (\lambda + \gamma_{1} )}} \hfill \\ \end{aligned} \right)$$

39 $$p_{66}^{5} = \frac{\lambda }{{(\lambda + \beta_{1} )}}\left( \begin{aligned} g*(\gamma_{2} ) - g*(\lambda + \beta_{1} + \gamma_{2} ) \hfill \\ \, \hfill \\ \end{aligned} \right)$$

40 $$p_{66}^{(7,3,5)} = \lambda \gamma_{2} \gamma_{1} \left( \begin{aligned} &\frac{{g*(\lambda + \beta_{1} + \gamma_{2} )}}{{\left( {\lambda - \gamma_{2} + \gamma_{1} } \right)}}\left( {\frac{1}{{\left( {\beta_{1} + \gamma_{2} - \gamma_{1} } \right)^{2} }} - \frac{1}{{(\lambda + \beta_{1} )^{2} }}} \right) - \frac{{g*^{\prime } (\lambda + \beta_{1} + \gamma_{2} )}}{{\left( {\beta_{1} + \gamma_{2} - \gamma_{1} } \right)(\lambda + \beta_{1} )}} \hfill \\ &- \frac{{g*(\lambda + \gamma_{1} )}}{{\left( {\beta_{1} + \gamma_{2} - \gamma_{1} } \right)^{2} (\lambda + \gamma_{1} - \gamma_{2} )}} + \frac{{g*(\gamma_{2} )}}{{\left( {\beta_{1} + \lambda } \right)^{2} (\lambda + \gamma_{1} - \gamma_{2} )}} \hfill \\ \end{aligned} \right)$$

41 $$p_{68}^{9} = \frac{{\beta_{1} }}{{(\lambda + \beta_{1} + \gamma_{2} )}}(1 - g*(\lambda + \beta_{1} + \gamma_{2} ))$$

42 $$p_{68}^{(7,3,9)} = \beta_{1} \gamma_{2} \gamma_{1} \left( \begin{aligned} &\frac{{g*(\lambda + \beta_{1} + \gamma_{2} )}}{{\left( {\lambda + \gamma_{1} } \right)}}\left( {\frac{1}{{\left( {\beta_{1} + \gamma_{2} - \gamma_{1} } \right)^{2} }} - \frac{1}{{(\lambda + \beta_{1} )^{2} }}} \right) - \frac{{g*^{\prime } (\lambda + \beta_{1} + \gamma_{2} )}}{{\left( {\beta_{1} + \gamma_{2} - \gamma_{1} } \right)(\lambda + \beta_{1} + \gamma_{2} )}} \hfill \\ &- \frac{{g*(\lambda + \gamma_{1} )}}{{\left( {\beta_{1} + \gamma_{2} - \gamma_{1} } \right)^{2} (\lambda + \gamma_{1} )}} + \frac{1}{{\left( {\beta_{1} + \lambda + \gamma_{2} } \right)^{2} (\lambda + \gamma_{1} )}} \hfill \\ \end{aligned} \right)$$

43 $$p_{80} = 1$$

44 $$\begin{aligned} &\mu_{0} = \frac{1}{{\left( {\lambda + \gamma_{1} } \right)}},\,\,\mu_{1} = \frac{1}{{\left( {\lambda + \gamma_{1} } \right)}}(1 - g*(\lambda + \gamma_{1} )),\,\,\mu_{2} = \frac{1}{{\left( {\lambda + \beta_{1} + \gamma_{2} } \right)}}, \hfill \\ &\mu_{6} = \frac{{\left( {1 - g^{*} \left( {\lambda + \beta_{1} + \gamma_{2} } \right)} \right)}}{{\left( {\lambda + \beta_{1} + \gamma_{2} } \right)}},\;\mu_{8} = \frac{1}{{\gamma_{2} }} \hfill \\ \end{aligned}$$

## Results and discussion

Numerous graphs have been plotted for the availability and the profit with respect to rates/revenue per unit up time for different values of rates/costs. The values of other parameters are given in Table 1. The following interpretations can be made from the graphs.

**Table 1:** Comparison table

Figure no.	Fixed parameter	Comparison with respect to	Which model is better (according to different situations)		
			Model 1 is better if	Model 2 is better if	Both the models are equally good
4	γ_1_ = 0.008/hr, γ_2_ = 0.235/hr, γ_3_ = 0.353/hr, γ_4_ = 0.4213/hr, α = 0.05/hr, β_1_ = 0.002/hr, p_1_ = 0.665, p_2_ = 0.335, C_2_ = INR 500, C_1_ = INR 700, C_3_ = INR 400, ICA = INR 500, λ = 0.003/hr	Availability (AD) when d ≥ p	λ > 0.3903	λ < 0.3903	λ = 0.3903
5		Profit			
		C_4_ = 100	C_0_ < 728.173	C_0_ > 728.173	C_0_ = 728.173
		C_4_ = 600	C_0_ < 574.291	C_0_ > 574.291	C_0_ = 574.291
		C_4_ = 1100	C_0_ < 352.178	C_0_ > 352.178	C_0_ = 352.178
6	λ = 0.003/hr, α = 0.05/hr, γ_1_ = 0.008/hr, γ_2_ = 0.23/hr, γ_3_ = 0.353/hr, γ_4_ = 0.4213/hr, β_1_ = 0.002/hr, p_1_ = 0.665, p_2_ = 0.335, C_0_ = INR 7000, C_1_ = INR 700, C_4_ = INR 400, ICA = INR 500	Profit			
		C_3_ = 200	C_2_ > 574.248	C_2_ < 574.248	C_2_ = 574.248
		C_3_ = 10,200	C_2_ > 478.789	C_2_ < 478.789	C_2_ = 478.789
		C_3_ = 20,200	C_2_ > 391.432	C_2_ < 391.432	C_2_ = 391.432
7	λ = 0.003/hr, α = 0.05/hr, γ_2_ = 0.235/hr, γ_3_ = 0.353/hr, γ_4_ = 0.4213/hr, β_1_ = 0.002/hr, p_1_ = 0.665, p_2_ = 0.335, C_2_ = INR 500, C_0_ = INR 7000, C_3_ = INR 400, C_4_ = INR 400, ICA = INR 500, γ_1_ = 0.008/hr	Profit	C_1_ < 202.594	C_1_ > 202.594	C_1_ = 202.594
8	λ = 0.003/hr, α = 0.05/hr, γ_1_ = 0.008/hr, γ_2_ = 0.235/hr, γ_3_ = 0.353/hr, γ_4_ = 0.4213/hr, β_1_ = 0.002/hr, p_1_ = 0.665, p_2_ = 0.335, C_2_ = INR 500, C_0_ = INR 7000, C_3_ = INR 400, C_4_ = INR 400, C_1_ = INR 700	Profit	ICA > 1632.46	ICA < 1632.46	ICA = 1632.46

It has been observed that the MTSF for the Model 2 is greater than that of Model 1 irrespective of the values of failure rate (λ). However, availability of one may be greater or lesser than that of the other depending upon the values of λ as discussed below.

**Fig. 4:**
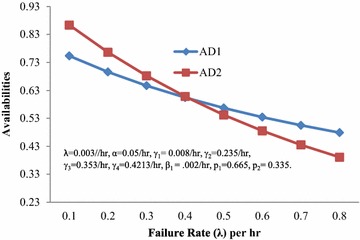
Availabilities (*AD1*,* AD2*) when demand ≥ production versus failure rate (λ)

Figure 4 depicts the behaviour of the availabilities (AD1, AD2) when demand is not less than the production with respect to the failure rate (λ). It can be interpreted from the graph that AD2 is < or = or >AD1 according as λ> or = or <0.3903.

So far as the behaviour of the availabilities (AP1, AP2) when demand is less than the production with respect to the failure rate (λ) is concerned, it has been observed that Model 1 is better than that of Model 2, whatever the values of λ may be.

**Fig. 5:**
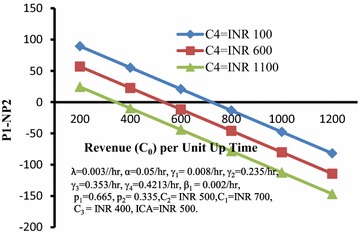
Difference between profits (*P1-NP2*) versus revenue (*C*_*0*_) per unit up time for different values of loss (*C*_*4*_)

Figure 5 depicts the behaviour of the differences of profits (P1-NP2) with respect to revenue (C_0_) per unit up time for different values of loss (C_4_) per unit time during the system remains down. It can be seen that this difference decreases with increase in the values of C_0_ and has lower values for higher C_4_.

**Fig. 6:**
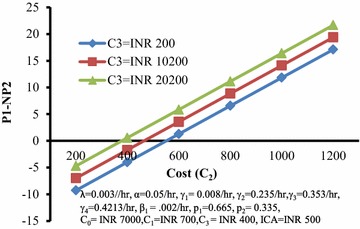
Differences of profits (*P1-NP2*) versus cost (*C*_*2*_) for different values of cost (*C*_*3*_)

Figure 6 depicts the behaviour of the differences of profits (P1-NP2) with respect to cost (C_2_) for different values of cost (C_3_). It can be seen that this difference increases with increase in the values of C_2_ and has higher values for higher C_3_.

**Fig. 7:**
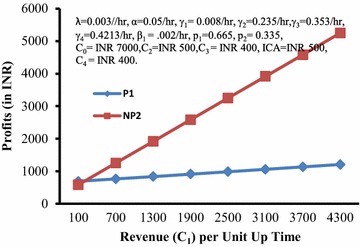
Profits (*P1*,* NP2*) versus revenue (*C*_*1*_) per unit up time

Figure 7 depicts the behaviour of profits (P1, NP2) with respect to revenue (C_1_) per unit up time. It is clear from the graph that NP2> or = or <P1 according as C_1_ is > or = or <202.594.

**Fig. 8:**
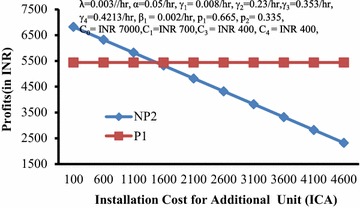
Profits (*P1*,* NP2*) versus Installation cost for additional unit (*ICA*)

Figure 8 depicts the behaviour of profits (P1, NP2) with respect to installation cost (ICA) for additional unit. It is clear from the graph that NP2> or = or <P1 according as ICA is < or = or > 1632.46.

### Comparative analysis between Model 2 and the Model discussed in Malhotra and Taneja (2014)

Here, we wish to report the comparison between the Model 2 and that discussed in Malhotra and Taneja (2014) also as under:

1. MTSF in case of Model 2 is greater than that of the earlier paper, irrespective of the value of λ. However, availability in case of the former is greater or lesser than that the latter according as the demand is lesser or greater than production.
2. So far as the profit of the system is concerned, Model 2 is better or worse than that discussed in Malhotra and Taneja (2014) according as the value of revenue per unit up time is lesser or greater than that at cut-off point as shown in Fig. 9.

**Fig. 9:**
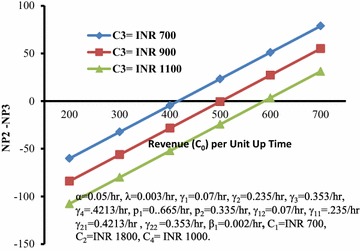
Differences between profits (*NP2-NP3*) versus revenue (*C*_*0*_) per unit up time for different values of cost (*C*_*3*_)
3. Behaviour of the profit with respect to loss per unit down time reveals that Model discussed in Malhotra and Taneja (2014) is better or worse than Model 2 if the loss per unit down time is greater or lesser than the value at cut-off point as shown in Fig. 10.

**Fig. 10:**
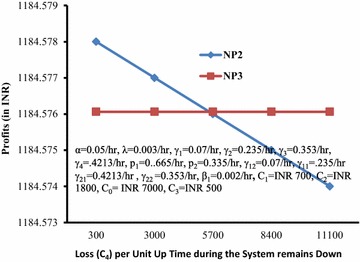
Profits (*NP2*,* NP3*) versus loss (*C*_*4*_) per unit time during the system remains down

## Conclusion

The semi-Markov process is applied to show the comparison of two stochastic models of a cable manufacturing plant with varying demand. The comparison of systems is done by means of MTSF, steady state availabilities and profit function.

In the terms of availability (when demand is not less than production), Model 2 is more profitable than Model 1, provided the failure rate does not exceed the calculated cut-off value. It can also be observed that all the availabilities decrease with increase in the value of failure rate. The lower limits (cut-off points) for the revenue per unit up time (when demand is not less than the production; when demand is less than production) for positive profit have been obtained, which may be quite useful for the system manufacturers/engineers/system analysts to check which model is best. Cut-off points for cost of engaging the repairman for repair and installation cost for additional unit has also been obtained. If these costs exceed corresponding cut-off values, Model 1 should be preferred.

Thus cut-off points on taking various numerical values for different parameters (rates/costs) prove to be helpful in taking important decisions so far as the reliability and the profitability of the system is concerned. These also help to conclude that which model gives more profit as compared to other under favourable conditions.

Which and when one model is better than the other has been presented in the Table 1.

The comparison between Model 2 and the model discussed in Malhotra and Taneja (2014) also reveals that none of these is always better than the other. One is better than the other for some values of failure rate/loss/revenue and worse for other values of these parameters.

## References

[CR1] Alidrisi MM (1992). The reliability of a dynamic warm standby redundant system of n components with imperfect switching. Microelectron Reliab.

[CR2] Chandrasekhar P, Natarajan R, Yadavalli VSS (2004). A study on a two-unit standby system with Erlangian repair time. Asia-Pac J Oper Res.

[CR3] Dessie (2014) Modeling of HIV/AIDS dynamic evolution using non-homogeneous semi-markov process. SpringerPlus 3:537 10.1186/2193-1801-3-537 PMC4175685 25279328

[CR4] Ke JC, Chu YK (2007). Comparative analysis of availability for a redundant repairable system. Appl Math Comput.

[CR5] Malhotra R, Taneja G (2013). Reliability and availability analysis of a single unit system with varying demand. Math J Interdiscip Sci.

[CR6] Malhotra R, Taneja G (2013). Reliability modelling of a cable manufacturing plant with variation in demand. Int J Res Mech Eng Technol.

[CR7] Malhotra R, Taneja G (2014) Stochastic analysis of a two-unit cold standby system wherein both units may become operative depending upon the demand. J Qual Reliab Eng. doi:10.1155/2014/896379**(Article ID 896379)**

[CR8] Malhotra R, Taneja G (2015). Comparative analysis of two single unit systems with production depending on demand. Ind Eng Lett.

[CR9] Mokaddis GS, Labib SW, El-Said KM (1994). Two models for two dissimilar-unit standby redundant system with three types of repair facilities and perfect or imperfect switch. Microelectron Reliab.

[CR10] Pan JN (1997). Reliability prediction of imperfect switching systems subject to multiple stresses. Microelectron Reliab.

[CR11] Taneja G, Malhotra R (2013). Cost-benefit analysis of a single unit system with scheduled maintenance and variation in demand. J Math Stat.

[CR12] Taneja G, Naveen V (2003). Comparative study of two reliability models with patience time and chances of non-availability of expert repairman. Pure Appl Math Sci LVII.

[CR13] Tuteja RK, Arora RT, Taneja G (1991). Analysis of a two-unit system with partial failures and three types of repairs. Reliab Eng Syst Saf.

[CR14] Wang KH, Chen YJ (2009). Comparative analysis of availability between three systems with general repair times, reboot delay and switching failures. Appl Math Comput.

[CR15] Xu H, Guo W, Yu J, Zhu G (2005). Asymptotic stability of a repairable system with imperfect switching mechanism. Int J Math Math Sci.

[CR16] Yusuf I (2014). Comparative analysis of profit between three dissimilar repairable redundant systems using supporting external device for operation. J Ind Eng Int.

[CR17] Zhang Z, Gao W, Zhou Y, Zhang Z (2012). Reliability modelling and maintenance optimization of the diesel system in locomotives. Maint Reliab.

